# ASC-J9® suppresses prostate cancer cell proliferation and invasion via altering the ATF3-PTK2 signaling

**DOI:** 10.1186/s13046-020-01760-2

**Published:** 2021-01-04

**Authors:** Hao Tian, Fu-ju Chou, Jing Tian, Yong Zhang, Bosen You, Chi-Ping Huang, Shuyuan Yeh, Yuanjie Niu, Chawnshang Chang

**Affiliations:** 1 Department of Urology, Tianjin Institute of Urology, The Second Hospital of Tianjin Medical University, Tianjin Medical University, Tianjin, 300211 China; 2 George Whipple Lab for Cancer Research, Departments of Pathology and Urology, University of Rochester Medical Center, Rochester, NY 14642 USA; 3 Department of Urology, the Second Hospital of Hebei Medical University, Shijiazhuang, 050000 China; 4 Sex Hormone Research Center, Department of Urology, China Medical University, Taichung, 404 Taiwan

**Keywords:** ASC-J9®, ATF3, Prostate cancer, ATF3 response element, PTK2

## Abstract

**Background:**

Early studies indicated that ASC-J9®, an androgen receptor (AR) degradation enhancer, could suppress the prostate cancer (PCa) progression. Here we found ASC-J9® could also suppress the PCa progression via an AR-independent mechanism, which might involve modulating the tumor suppressor ATF3 expression.

**Methods:**

The lentiviral system was used to modify gene expression in C4–2, CWR22Rv1 and PC-3 cells. Western blot and Immunohistochemistry were used to detect protein expression. MTT and Transwell assays were used to test the proliferation and invasion ability.

**Results:**

ASC-J9® can suppress PCa cell proliferation and invasion in both PCa C4–2 and CWR22Rv1 cells via altering the ATF3 expression. Further mechanistic studies reveal that ASC-J9® can increase the ATF3 expression via decreasing Glutamate-cysteine ligase catalytic (GCLC) subunit expression, which can then lead to decrease the PTK2 expression. Human clinical studies further linked the ATF3 expression to the PCa progression. Preclinical studies using in vivo mouse model also proved ASC-J9® could suppress AR-independent PCa cell invasion, which could be reversed after suppressing ATF3.

**Conclusions:**

ASC-J9® can function via altering ATF3/PTK2 signaling to suppress the PCa progression in an AR-independent manner.

**Supplementary Information:**

The online version contains supplementary material available at 10.1186/s13046-020-01760-2.

## Background

In the United States, prostate cancer (PCa) is the 2nd most fatal tumor in males, with an estimated 174,650 new cases and 31,620 deaths in 2019 [1]. The current standard therapy remains the androgen deprivation therapy (ADT), which was established in 1940 [2]. However, most PCa patients receiving the ADT relapse after 1–2 years of treatment with the development of castration-resistant PCa (CRPC) [3]. While the recently developed antiandrogen Enzalutamide (Enz) could extend patients survival by 4.8 months [4], eventually it also fails with the development of antiandrogen resistance with some adverse effects [5–10].

Recent studies indicated several small molecules might be able to target the androgen receptor (AR) to suppress the PCa progression, unlike the current antiandrogens that suppress androgens from binding to the AR [11–17]. Among these small anti-AR molecules, the ASC-J9® was the first identified AR degradation enhancer that could degrade AR protein to suppress PCa cell proliferation and invasion [6, 16–21]. Since ASC-J9® can suppress PCa cell invasion, which contrasts with the current antiandrogens Casodex or Enz that increase the PCa cell invasion in multiple PCa cells [9, 22–25], it will be interesting to see if ASC-J9® can also function via a non-AR mechanism to suppress the PCa cell invasion.

Here we found ASC-J9®, and not the Enz or AR-shRNA, might function via increasing the expression of Activating Transcription Factor 3 (ATF3) to suppress the PCa cell proliferation and invasion. As a transcription factor, ATF3 belongs to the CREB family [26], may play important roles in inflammation [27], and can also function as a tumor suppressor via altering cellular stress in multiple tumors [28, 29]. For example, ATF3 can suppress bladder cancer and esophageal cancer growth and invasion [29, 30]. As downstream of reactive oxygen species (ROS), ATF3 can also mediate ROS-induced cell apoptosis in human colorectal cancer cells and lung cancer [31–33]. Although most studies indicated ATF3 could inhibit cancer cell growth via increasing apoptosis in multiple cancers, other groups found that ATF3 may increase cancer cell proliferation [34–38]. These contrasting results suggest that further studies may be needed to demonstrate its impact on the PCa progression. We also identified a non-AR pathway mediated by ASC-J9® and investigated the mechanism of function by in vivo and in vitro assays.

## Materials and methods

### Cell culture

PCa cells C4–2, CWR22Rv1, and PC3 were cultured in RMPI 1640 media containing 10% fetal bovine serum, 2 mM L-glutamine, 100 μg/ml streptomycin, and 100 units/ml penicillin. HEK293T cell line was cultured in DMEM media containing 10% fetal bovine serum, 2 mM L-glutamine, 100 μg/ml streptomycin, and 100 units/ml penicillin. All cell lines were purchased from the American Type Culture Collection (ATCC, Manassas, VA, USA) and tested to be bacteria and mycoplasma free according to the relevant instructions and cultured in humidified 5% carbon-dioxide cell incubator.

### Lentivirus prepartion

The shATF3 was constructed into pLKO.1 lentiviral vector and oeATF3 and oePTK2 were constructed into pWPI lentiviral vector. These plasmids were co-transfected into the 293 T cells with the packaging and envelope plasmids psPAX2 and pMD2G by the standard calcium chloride transfection (lentivirus:packaging plasmid:envelope plasmid = 2:1:1). After 48 h, lentiviral supernatants were collected and concentrated by ultracentrifugation or PEG4000+centrifugation and stored at − 80 °C.

### Quantitative real time-PCR and RNA-sequence

Total RNA was isolated using Invitrogen Trizol reagent (#15596026, Thermofisher, Waltham, MA, USA) according to the manufactures’ instructions. The isolated total RNA was then reverse transcribed into cDNA using the iScriptTM cDNA Synthesis Kit (#1708891, Bio-Rad, Hercules, CA, USA). In the Bio-Rad CFX96 system, quantitative real-time PCR (qRT-PCR) was performed with iQTM SYBR® Green Supermix (#1708880, Bio-Rad) to determine the mRNA expression level of the gene of interest. Expression levels were normalized to GAPDH expression and expression levels were calculated by the 2^−ΔΔCt^ method. For RNA-sequencing, RNA was submitted to the University of Rochester Genomics Research Center. The expression of genes of interest were analyzed and compared between control and treatment groups. The candidate genes were further confirmed by qRT-PCR.

### Western blot

The concentration of protein lysates was measured by the BSA method, the sample (50 μg/lane) was added and separated by electrophoresis in 10% SDS/PAGE gel, then transferred to a PVDF membrane, and after blocking with 5% non-fat milk for 1 h, they were appropriately incubated with diluted specific anti-ATF3 (#SC188, Santa Cruz, Dallas, TX, USA), PTK2 (#SC271126, Santa Cruz), or GAPDH (#SC166, Santa Cruz) at 4 °C overnight, then incubated with HRP-conjugated secondary antibody for 1 h at room temperature. The antigen-antibody complex was incubated with West Femto Maximum Sensitivity Substrate (# 34095, ThermoFisher Scientific) and signal was detected by Bio-Rad imaging system.

### Analysis of ROS levels

C4–2 and CWR22Rv1 cells were seeded in 24-well plates and cultured with RMPI1640 overnight. Then these cells were treated with 10 μM Dihydroethidium (DHE) for 1 h at 37 °C after being treated with ASC-J9® and/or 5 mM N-acetyl-L-cysteine (NAC) for 48 h, then the fluorescence images were captured by fluorescence microscope.

### Cell invasion assay

Matrigel was diluted with serum-free RMPI-1640 medium at 1:20, applied to the upper-chamber of 8 μm-pore-size polycarbonate membrane filters, and incubated at 37 °C for 6 h. PCa cells were collected, suspended with serum-free media, and plated into upper chambers at 100,000 cells/well. Then 750 μl RMPI-1640 media containing 10% FBS was added in the lower chambers for incubation for 24 h in 37 °C CO_2_ incubator. The cells on the membranes upper surface were removed with cotton swabs and the invasive cells attached to the bottom surface of membranes were fixed by 4% paraformaldehyde, stained with 1% crystal violet, and counted in five randomly chosen microscope fields (100X) for each membrane.

### Cell proliferation assay

20,000 cells were seeded in 24-well culture plates and allowed to attach overnight. After culturing for 0, 2, 4, and 6, days 400 μl of 5% 3-(4,5-dimethylthiazol-2-yl)-2,5-diphenyltetrazolium bromide (MTT) solution, which was buffered in PBS, was added to each well. Plates were incubated for 30 min at 37 °C. Then, the crystals were reconstituted by dilution with 400 ml DMSO. The absorbances at 570 nm were measured by microplate reader.

### Immunohistochemistry staining (IHC)

Tissues were fixed, embedded, and cut into 5 μm sections and placed onto slides. After deparaffinization, hydration, and antigen retrieval, each slide was incubated with endogenous peroxidase blocking solution and incubated with primary antibody at 4 °C overnight. The next day, after washing with PBS buffered saline, the slides were incubated for 60 min with biotin-conjugated secondary antibody (Vector Laboratories, Burlingame, CA, USA) at room temperature, and washed with PBS. VECTASTAIN ABC peroxidase system and 3, 3′-diaminobenzidine (DAB) kit (Vector Laboratories) was added for incubation for color detection. Finally, the slides were stained with haematoxylin.

### GSH assay

C4–2 and CWR22Rv1 Cells were untreated or treated with 5 μM ASC-J9® for 24 h. Cells were collected and washed by PBS, resuspended with 4-Morpholineethanesulfonic acid hydrate (MES) buffer, then sonicated, sedimented, and deproteinated with metaphosphoric acid and triethanolamine. Total GSH concentration was determined with the Glutathione Assay Kit (#703002, Cayman Chemical, Ann Arbor, MI, USA) according to the instructions of the manufacturer.

### Luciferase assay

The PTK2 promoter was constructed into pGL3-basal luciferase reporter vector (Promega, Madison, WI, USA) and transfected into C4–2 and CWR22Rv1 cells seeded in 24-well plates using Lipofectamine 3000 transfection reagent according to the manufacturer’s instructions. PRL-TK was used as an internal reference. After 48 h, luciferase activity was measured by the dual luciferase assay (Promega) according to the manufacturer’s manual.

### Chromatin immunoprecipitation assay (ChIP)

After cells were cross-linked with 4% formaldehyde, they were harvested, lysed, and sonicated to obtain a genomic DNA fragment from 100 bp to 300 bp. Then cell lysates were pre-cleared with normal rabbit IgG and protein A-agarose. Subsequently, 2 μg of anti-ATF3 antibody was added to the cell lysate and incubated overnight at 4 °C. IgG was used as a negative control for this reaction. Specific primers were designed to amplify the target sequence of the PTK2 promoter and PCR products were identified by agarose gel electrophoresis.

### In vivo mice studies

PC-3 cells were transduced with luciferase and with/without shATF3 for anterior prostate xenografts in 24 6–8 weeks old nude mice (National Cancer Institute, Bethesda, MD, USA). After anesthetizing nude mice with isoflurane (NDC 11695–0500-2, Henry Schein, Dublin, OH, USA), the prepared PC-3 cells at 1 × 10^6^ were mixed with Matrigel, 1:1 and were injected into anterior prostates of 3 groups of mice. After tumors developed (~ 2 weeks) the mice were treated with/without ASC-J9® as follows: 1) PC-3-Luc-scramble+vehicle; 2)) PC-3-Luc-scramble+ASC-J9®; 3)) PC-3-Luc-shATF3 + ASC-J9®. Tumor development and metastasis were monitored by Fluorescent Imager (IVIS Spectrum, Caliper Life Sciences, Hopkinton, MA, USA) every 2 weeks. ASC-J9® (75 mg/kg) was diluted in DMA, Cremophor EL (CrEL), and Saline (or the same volume of vehicle) for intraperitoneally injection into mice every other day (total 5 times) after tumor detected by IVIS. After 8–10 weeks, mice were euthanized and tumors and metastases removed for further study. All experiments were approved by the University of Rochester Medical Center and in accordance with the corresponding institutional guidelines.

### Statistical analysis

Quantitative real time-PCR Data are expressed as mean ± SEM from at least 3 independent experiments with data points performed in triplicate. Other Data are expressed as mean ± SD. Statistical analyses involved t-test and ANOVA with SPSS 17.0 (SPSS Inc., Chicago, IL, USA). *P* < 0.05 is considered statistically significant.

## Results

### ASC-J9®, and not Enz or AR-shRNA, increases tumor suppressor ATF3 expression

**Fig. 1:**
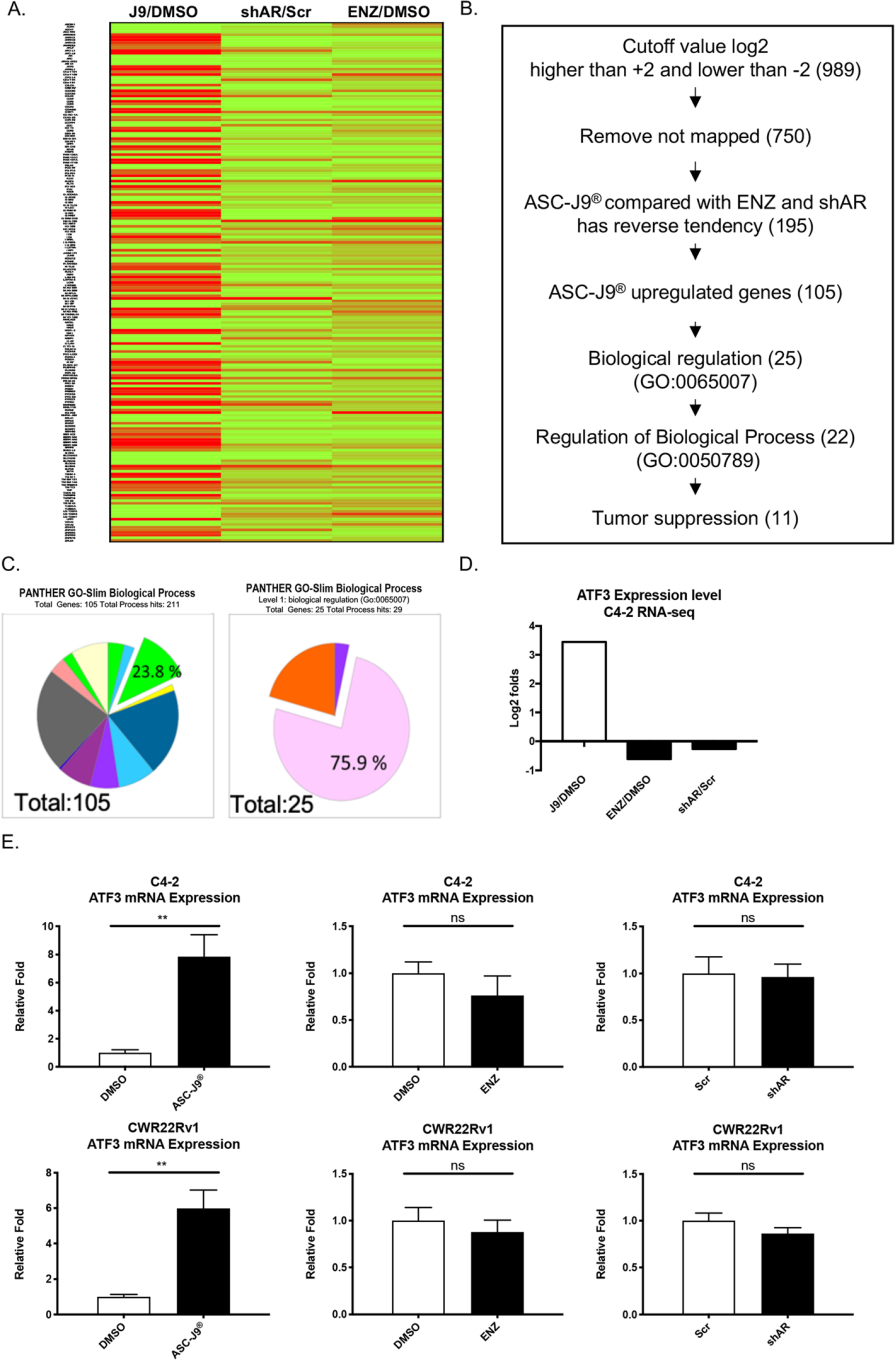
RNA-sequence analysis of ATF3 in C4–2. (A) C4–2 cells were treated with 5 μM ASC-J9® (or DMSO), or infected with shAR (or Scr), or 10 μM Enz (or DMSO), followed by RNA-seq. In the heat map, red lines are shown as high expressed genes, green lines are shown as low expressed genes, J9/DMSO = ASC-J9® vs. DMSO, shAR/scr = knockdown AR vs. scramble, ENZ/DMSO = Enz vs. DMSO. **b** The rationale of screening out the unique ASC-J9® tumor suppressor genes. **c** Functional analysis via gene ontology selected out 11 cancer suppression genes including ATF3. Left chart shows 105 ASC-J9® specifically upregulated genes GO analysis. Right chart shows 25 ASC-J9® specifically upregulated genes involved in Biological regulation. Different colors represent different Biological Process. The color labeled with percentage includes ATF3. **d** Isolating RNA expression data from RNA-seq (see **a**), ASC-J9® uniquely increases ATF3 expression compared with Enz and shAR groups. **e** In C4–2 (upper panels) and CWR22Rv1 (lower panels) cells, qRT-PCR results reveal ATF3 expression level in 5 μM ASC-J9® treatment group was significantly higher than 10 μM Enz treatment and shAR transduced group. Data represent the Mean ± SEM, **p < 0.01, ns = no significance, by Unpaired t-test

Early studies indicated that ASC-J9® could suppress PCa cell proliferation and invasion via degrading the AR [16, 18, 19, 21, 26, 39, 40]. Its linkage to the suppression of PCa progression via a non-AR mechanism, however, remains to be further elucidated. Using ASC-J9® vs Enz or AR-shRNA, we first applied the RNAseq assay (Fig. 1a) to compare their differential effects on their downstream genes regulation in the PCa C4–2 cells (Fig. 1d). The results revealed that among 195 mapped genes, 105 genes are only increased by ASC-J9®, and not Enz and AR-shRNA, with 23.8% of these ASC-J9® elevated genes have biological regulation (Fig. 1b). Results from GO analysis further revealed that among these ASC-J9® elevated genes, only 11 genes, including ATF3, are responsible for the tumor suppression (Fig. 1b and c). Importantly, results from qRT-PCR assay further confirmed that only ASC-J9®, not Enz or AR-shRNA, could increase ATF3 expression (Fig. 1e).

Together, these contrasting results among those anti-AR molecules, including ASC-J9®, Enz, and AR-shRNA, suggest that ASC-J9® may also be able to function via a non-AR mechanism to regulate the ATF3 expression.

### ASC-J9®-increased ATF3 expression led to decrease the PCa cell proliferation and invasion in the PCa C4–2 and CWR22Rv1 cells

**Fig. 2:**
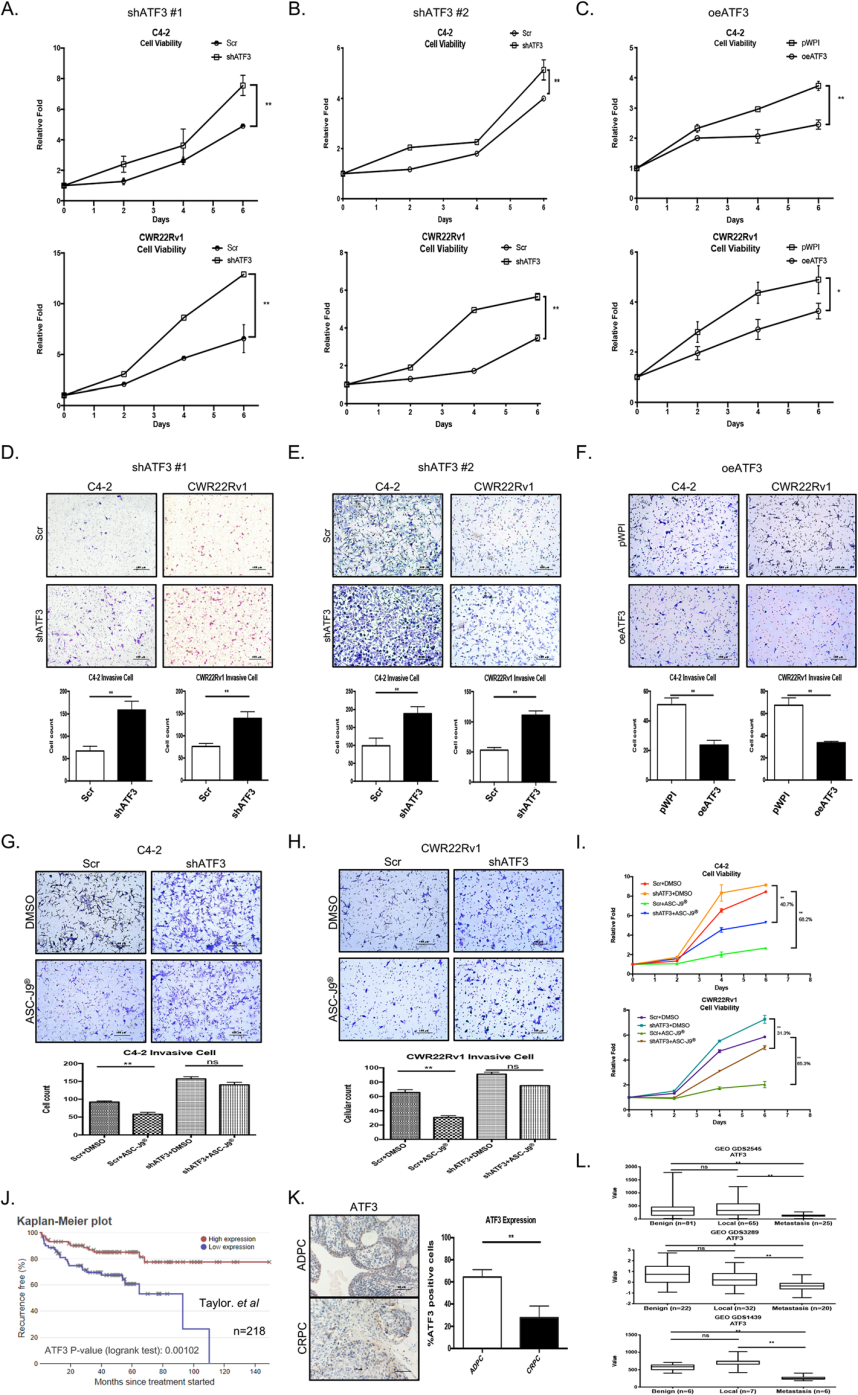
ATF3 regulates invasion and proliferation in PCa cells (**a**-**b**) MTT assay using 2 different shATF3s (shATF3 #1 and shATF3 #2) indicate suppressing ATF3 could increase cell growth in C4–2 cells (upper) and in CWR22Rv-1 cells (lower). **c** MTT assay indicates oeATF3 in C4–2 (upper) and CWR22Rv1 (lower) cells reduces cell growth. **d**-**e** Invasion assay shows using shATF3 #1 and shATF3 #2 to knock down ATF3 increases cell invasion in C4–2 (right) and CWR22Rv1 (left) cells. **f** Overexpression of ATF3 (oeATF3) in C4–2 (left) and CWR22Rv1 (right) can decrease cell invasion. **g**-**h** Transwell assays show that ASC-J9® could suppress PCa invasion via increased ATF3, then adding shATF3 could interrupt ASC-J9® effect to further increase invasive cells in C4–2 (**g**) and CWR-22Rv1 (**h**) cells. **i** MTT analysis shows knock down of ATF3 in C4–2 and CWR22Rv1 cells reversed ASC-J9® treatment effects. **j** Kaplan-Meier plot demonstrates high ATF3 shows higher recurrence free percentage. **k** IHC from patients’ samples show ATF3 expression in ADPC (early stage) is higher than CRPC (late stage) patients, magnification, X200. **l** GEO dataset t analysis (GDS2545, S1439, S3289) indicate ATF3 expression decreases with progression of malignancy. For d-g and k, quantitations are below or right of images. Data represent the mean ± SD in (A-I). *p < 0.05, **p < 0.01, ns = not significant by Student t test or one-way ANOVA)

To investigate the consequences of ASC-J9®-increased ATF3 expression, we knocked down ATF3 expression via lentiviral infection using ATF3-shRNA-1 (shATF3 #1) and ATF3-shRNA-2 (shATF3 #2) (Supplementary Fig. 1). Results revealed that suppressing ATF3 expression with shATF3 #1 increased the PCa cell proliferation in both C4–2 and CWR22Rv1 cells (Fig. 2a). Similar results were also obtained when we replaced the shATF3 #1 with shATF3 #2 (Fig. 2b). In contrast, increased ATF3 expression via lentiviral infection of cDNA (oeATF3) can then lead to decrease the PCa cell proliferation in both C4–2 and CWR22Rv1 cells (Fig. 2c).

Similar results were also obtained when we replaced cell proliferation assay with a cell invasion assay, showing decreasing the ATF3 expression with two different ATF3-shRNAs led to increase the PCa cell invasion in both C4–2 and CWR22Rv1 cells (Fig. 2d-e), and increasing the ATF3 expression with oeATF3 led to inhibit the PCa cell invasion in both C4–2 and CWR22Rv1 cells (Fig. 2f).

Importantly, results from an interruption approach revealed that adding ATF-shRNAs could then partially reverse/block the ASC-J9®-suppressed PCa cells invasion (Fig. 2g-h) and cell proliferation (Fig. 2i) in both C4–2 and CWR22Rv1 cells.

Also, knocking down ATF3 in AR-negative cell PC-3, we get similar results. Knocking down ATF3 in PC-3 can increase cells growth and invasion, and vice versa (Supplementary Fig. 2a-d). Low expression of ATF3 can reverse the ASC-J9®-suppressed PCa cells growth (Supplementary Fig. 2e).

Together, results from Fig. 2a-i and Supplementary Figure 2a-e suggest that ASC-J9® can function via increasing the expression of ATF3 tumor suppressor to decrease the PCa cell proliferation and invasion.

### Human clinical data links the expression of ATF3 to the PCa progression

To further strengthen the above conclusions with multiple in vitro cell lines showing ATF3 may play suppressor roles to impact the PCa progression, we then applied the human clinical sample survey via analysis of ATF3 expression in the human PCa samples. We first analyzed the Kaplan-Meier dataset reported by Taylor et al. [41] and found that the expression of ATF3 expression is significantly lower in the recurrent PCa samples (Fig. 2j). Results shown in Fig. 2k using IHC staining also revealed that ATF3 expression in androgen-sensitive prostate cancer (ADPC) patients as significantly higher than that found in the CRPC patients. Importantly, results from the analysis of three GEO datasets [42–44] also indicated that the expression of ATF3 mRNA is decreased with the progression of malignancy in the human PCa samples (Fig. 2l).

Together, results from multiple human clinical sample surveys (Fig. 2j-l) suggest that the expression of ATF3 is negatively linked to the PCa progression.

### Mechanism dissection of how ASC-J9® can increase ATF3 expression: via blocking the glutamate-cysteine ligase catalytic (GCLC) subunit in an AR-independent manner

To dissect the mechanism of how ASC-J9® can increase ATF3 expression, we hypothesized that ASC-J9® might function via increasing reactive oxygen species (ROS) levels to increase the ATF3 expression since early studies indicated ATF3 might function via inducing ROS to alter the apoptosis in both tumor and normal cells [34, 45]. Recent studies also indicated that ASC-J9® could alter apoptosis and ROS in multiple cancer cells [46–48].

**Fig. 3:**
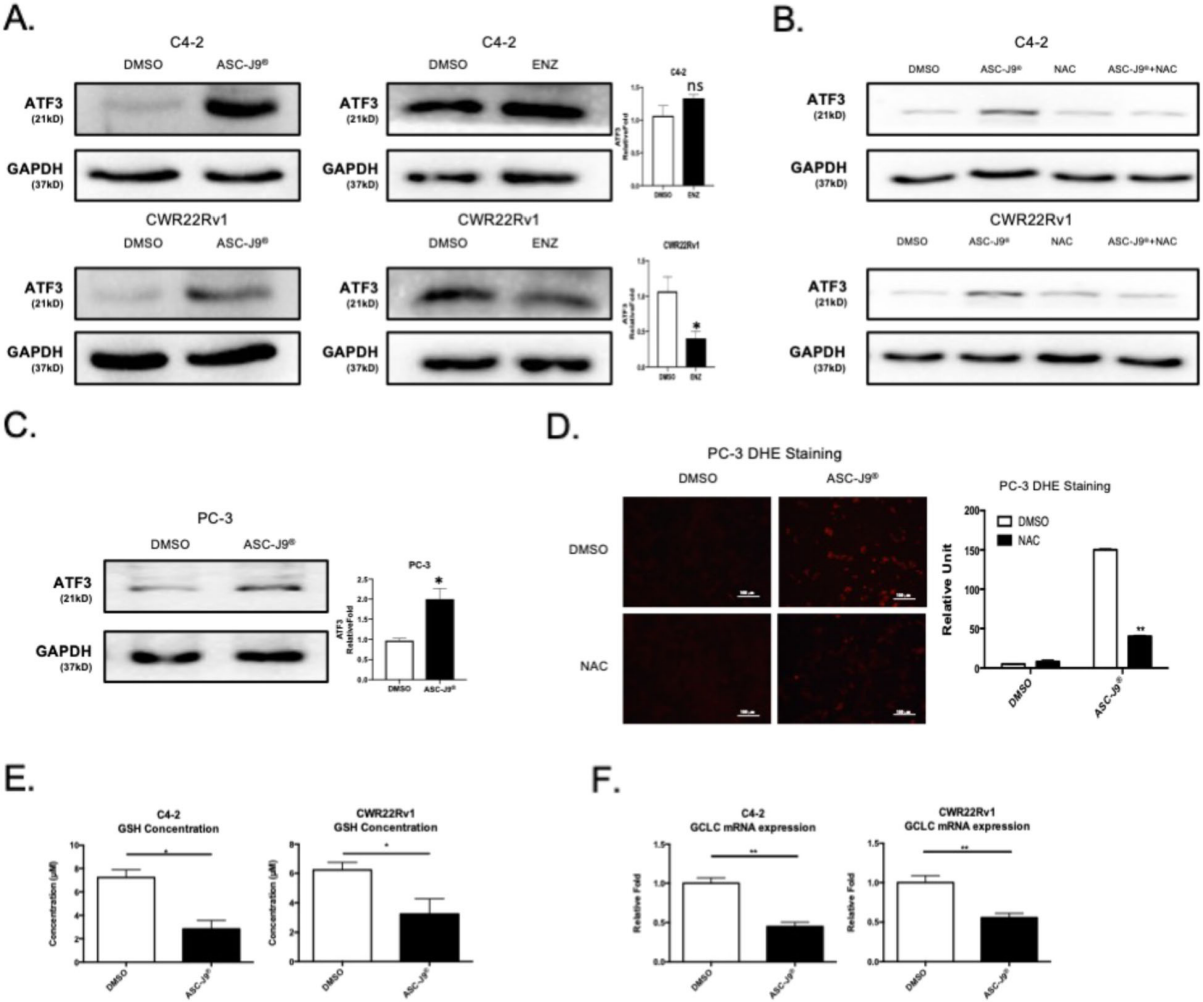
ASC-J9® increases ROS and decreases GCLC levels in PCa cells. **a** Western Blots show higher ATF3 expression with ASC-J9® treatment (left panels) than with Enz treatment (right panels) in C4–2 (upper) and CWR22Rv1 (lower) cells. **b** Western Blots show 5 mM NAC treatment can suppress ASC-J9® induced ATF3 expression in C4–2 (upper) and CWR22Rv1 (lower) cells. **c** Western Blot shows ASC-J9® induced ATF3 expression in AR negative PCa PC-3 cells. **d** DHE staining assay shows ROS level increase in PC3 after ASC-J9® treatment and 5 mM NAC treatment could suppress DHE signal. **e** GSH assay to reveal ASC-J9® can decrease GSH concentration in C4–2 and CWR22Rv1 cells. **f** The qRT-PCR of GSH upstream genes GCLC was decreased significantly in C4–2 and CWR22Rv1 cells when treated with ASC-J9®. For **a**, **c-d**, quantification is at the right. Data represent the mean ± SD except quantitative real time-PCR represent the mean ± SEM. *p < 0.05, **p < 0.01, ns = not significant, by unpaired Student t test

As expected, we first found that treating with ASC-J9®, and not Enz, could increase ATF3 expression at both mRNA (see Fig. 1d) and protein levels in both C4–2 and CWR22Rv1 cells (Fig. 3a). Treating C4–2 and CWR22Rv1 cell lines with N-acetylcysteine (NAC), the ROS inhibitor, could then block the ASC-J9® effects in these cells (Fig. 3b). Importantly, ASC-J9® also can increase ATF3 expression significantly in AR-negative PC-3 cells (Fig. 3c). NAC was visualized, using DHE staining, which show that it could also block ASC-J9® effects in PC-3 cells (Fig. 3d). These results suggest ASC-J9® can suppress the PCa cell proliferation and invasion via altering ROS to increase ATF3 expression.

We then further studied whether ASC-J9® can influence the Glutathione-ascorbate cycle like Curcumin [49], and found treating with ASC-J9® led to decrease significantly the GSH concentration in C4–2, CWR22Rv1 cells (Fig. 3e) and in PC-3 cells (Supplementary Fig. 2f), which is due to ASC-J9® decreasing the Glutamate-cysteine ligase catalytic (GCLC) subunit expression (Fig. 3f, Supplementary Fig. 2g) in these three PCa cells.

Together, results from Fig. 3a-f and Supplementary Figure. 2f-g suggest that ASC-J9® can increase ATF3 expression by altering the GCLC in an AR-independent manner.

### Mechanism dissection of why ASC-J9®-increased ATF3 can suppress PCa cell proliferation and invasion: via suppressing the PTK2

**Fig. 4:**
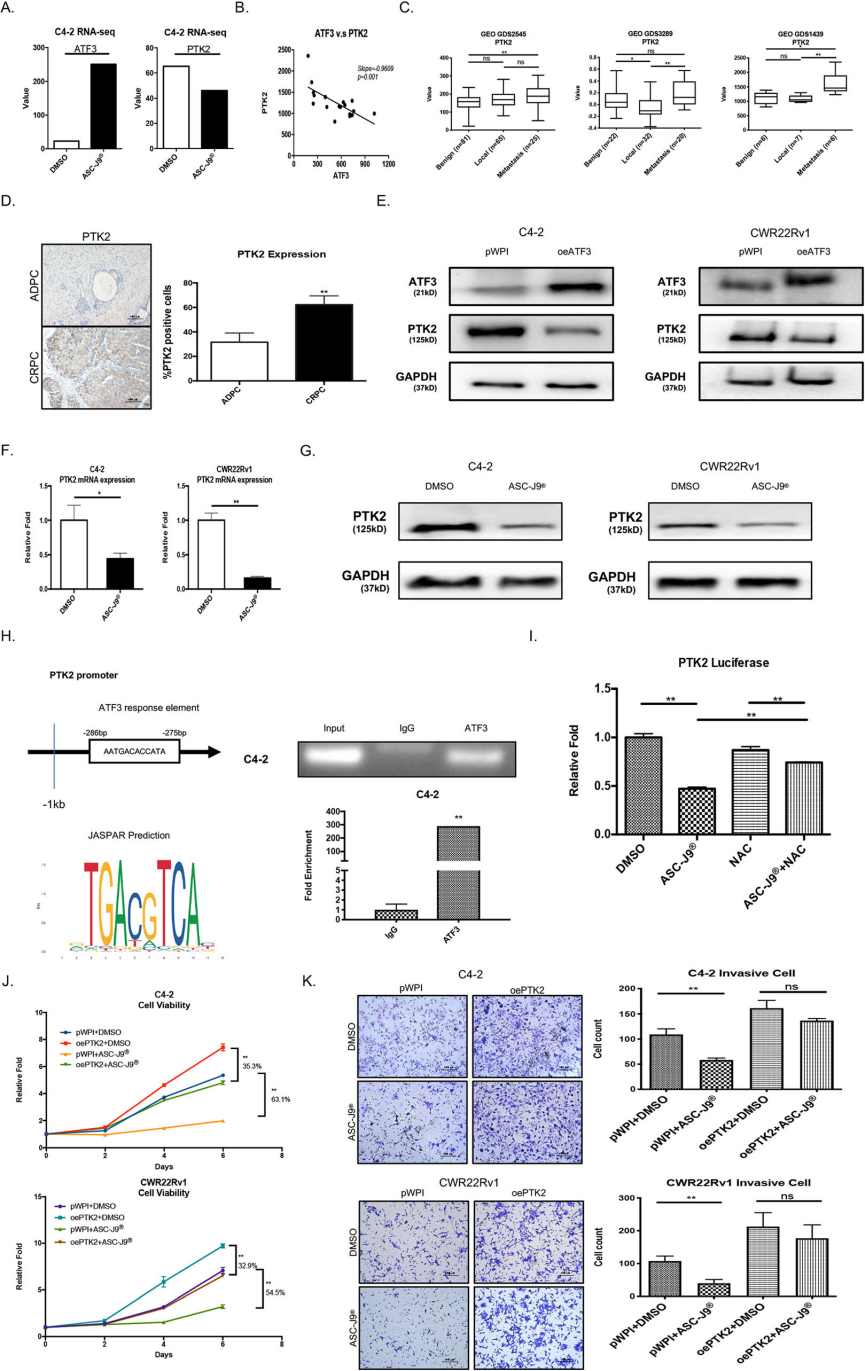
ATF3 suppresses PTK2 expression in PCa cells (**a**) RNA-Sequencing shows the ATF3 and PTK expression with reverse tendency under ASC-J9®^.^ treatment in C4–2 cells. **b** The linear regression shows ATF3 and PTK2 with negative correlation based on GEO Database GDS1239. **c** PTK2 mRNA expression in three human prostate databases (GDS2545, GDS1439, and GDS3289) demonstrate late stage tumors have higher PTK2 expression. **d** IHC from clinical samples show PTK2 expression lower in ADPC (early stage) and higher in CRPC (late stage) tumors. **e** Overexpressed ATF3 (oeATF3) could suppress PTK2 expression in C4–2 (left) and CWR22Rv1 (right) cells. **f** qRT-PCR revealed ASC-J9® treatment could decrease PTK2 expression in C4–2 (left) and CWR22rv1 (right) cells. **g** Western Blots show ASC-J9® treatment could suppress PTK2 expression level in C4–2 (left) and CWR22Rv1 (right) cells. **h** The diagram (upper left) shows ATF3 response element that is predicted by JASPAR (lower left) in the promoter of PTK2 and the ATF3 response element. Chip-PCR (upper right) and Chip-qRT-PCR (lower right) show ATF3 can bind to PTK2’s promoter region in C4–2 cells. **i** Luciferase assays show ASC-J9® could suppress PTK2 expression level and NAC could partially reverse this suppression in C4–2 cells. (J-K) MTT (**j**) and Invasion assays (**k**) show that oePTK2 can partly reverse the oeATF3 and ASC-J9® treatment effects in C4–2 (upper) and CWR22Rv1 (lower) cells. Data represent the mean ± SD except qRT-PCR represent the mean ± SEM. *p < 0.05, **p < 0.01, ns = not significant, by unpaired Student t test

Next, to study why ASC-J9®-increased ATF3 can suppress PCa cell proliferation and invasion, we analyzed the potential ATF3 downstream targets via RNAseq assay, and results revealed the negative correlation showing the expression of PTK2, a key factor to promote the proliferation and invasion of cancer [50], is decreased with the increase of ATF3 (Fig. 4a), which is also in agreement with the result from correlation analysis of the database from Jacobson, J.R., et al. [51], showing the expression of ATF3 was negatively correlated with the expression of PTK2 (Fig. 4b).

Furthermore, results from 3 other databases [42–44] also indicated that the expression of PTK2 is linked positively to the PCa progression (Fig. 4c), and results of IHC (Fig. 4d) further confirmed that the expression of PTK2 in ADPC is significantly lower as compared to that in castration-resistant PCa (CRPC).

Importantly, increasing ATF3 via oeATF3 also led to decrease the PTK2 expression in the PCa C4–2, CWR22Rv1 cells (Fig. 4e) and PC-3 cells (Supplementary Fig. 3a), and increase ATF3 via adding ASC-J9® led to decrease the PTK2 expression in the C4–2 and CWR22Rv1 cells (Fig. 4f). Furthermore, treating with ASC-J9® increased ATF3 expression (see Fig. 3a) and decreased the expression of PTK2 in the C4–2, CWR22Rv1 and PC-3 cells (Fig. 4g, Supplementary Fig. 3b).

By Jaspar analysis, we found that there is an ATF3 binding site in the promoter region of PTK2 [52]. Further chip analysis was performed to confirm that ATF3 can bind to the promoter region of PTK2 (Fig. 4h). Results from Luciferase reporter assay further confirmed that ASC-J9® treatment can inhibit the expression of PTK2, while NAC treatment can partly block the ASC-J9®-inhibited PTK2 expression (Fig. 4i).

Finally, results from an interruption approach via increasing PTK2 in C4–2 and CWR22Rv1 cells also led to partially reverse the ASC-J9®-suppressed PCa cell proliferation and invasion (Fig. 4j-k).

Together, results from Fig. 4a-k and Supplementary Figure 3 suggest that ASC-J9®-increased ATF3 may function via altering the PTK2 expression to suppress the PCa cell proliferation and invasion.

### Preclinical studies using in vivo mouse model to confirm ASC-J9®/ATF3/PTK2 signaling can suppress PCa cell proliferation and invasion

To further prove all in vitro results in the in vivo mouse model, we orthotopically xenografted PCa cells. To minimize the AR effect, we chose AR negative PC3 cells and transfected them with luciferase reporter gene for weekly non-invasive in vivo imaging system (IVIS) monitoring and with/without shATF3. We xenografted treated cells into 24 6–8 weeks old mice anterior prostates divided into 3 treatment (vehicle or ASC-J9®) groups: 1) scramble with vehicle (Scr + Vehicle); 2) scramble with ASC-J9® (Scr + ASC-J9®); 3) ATF3-shRNA with ASC-J9® (shATF3 + ASC-J9®).

**Fig. 5:**
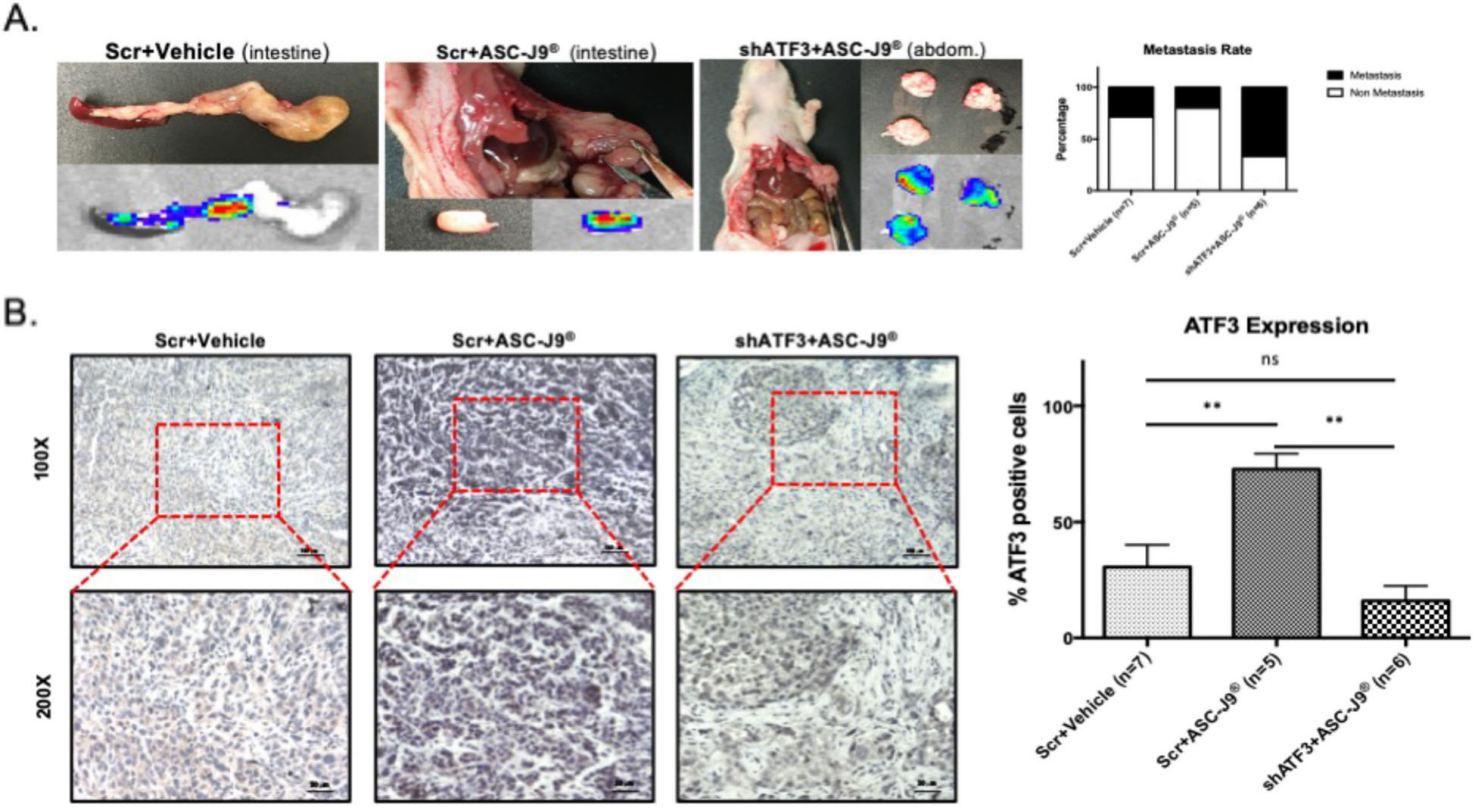
ASC-J9® can suppress tumor growth and metastasis via increasing ATF3 in mouse xenografts. **a** Anatomical pictures of intestinal/abdominal (left) and Metastasis statistics (right) show shATF3 treatment group have higher metastasis rates than Scr + Vehicle group and Scr + ASC-J9® group have lower rate than Scr + Vehicle group. **b** Representative IHC image (left) and quantification statistics (right) show ATF3 expression in mice xenograft, magnification, X100, X200. Data represent the mean ± SD, **p < 0.01, ns = not significant, by one-way ANOVA

After 8–10 weeks, the results revealed that the incidence of intestinal/abdominal metastasis in the Scr + ASC-J9® groups were relatively lower than that in the Scr + Vehicle group and importantly, the mice with ATF3-shRNAs had a reversal/blockage of the ASC-J9®-suppressed PCa cell invasion (Fig. 5a), suggesting ATF3 may play a tumor metastasis suppressor role.

IHC staining data for ATF3 suggested that ATF3 expression in PC-3 xenografted tumors was increased with ASC-J9® treatment (comparing Scr + Vehicle with Scr + ASC-J9®), and this increase was blocked/reversed after co-treatment of ASC-J9® with shATF3 (Fig. 5b). Meanwhile, knocking down ATF3 can reverse ASC-J9® inhibitory effects on PCa cell proliferation (Supplementary Fig. 4).

Together, results from Fig. 5 and Supplementary Figure 4 suggest that ASC-J9® can also suppress androgen-independent PCa cell invasion, and suppressing tumor suppressor ATF3 can increase AR-independent PCa cell invasion.

## Discussion

The purpose of PCa therapy is to eliminate primary tumors and prevent the recurrence and metastasis of tumors [53]. However, Enz treatment, as last line of medication to suppress the CRPC growth, may still result in Enz-resistance and increased metastasis [9, 22–25]. The evidence from knocking down AR suggest that AR may play an opposite role in the PCa cell proliferation vs cell invasion, and suppressing AR might increase the risk of PCa cell invasion [9, 22–25, 54]. Our study demonstrated the advantage of ASC-J9®, which not only suppressed PCa proliferation via degrading AR, but also activates ATF3 to suppress PCa invasion.

An early study demonstrated that ATF3 is mainly stimulated by ROS and plays an important role in the inflammatory response [55]. Other studies indicated that ATF3 could also increase progression in certain cancers [37, 38, 56]. However, our results found that ATF3 could play a suppressor role in the invasive ability of PCa cells. These controversial results suggest ATF3 may have different roles in different cancers, which might need further study.

Previously, our lab studies indicated that ASC-J9® treatment has an extra AR-independent function, which can suppress PCa cell proliferation via inhibiting STAT3 sumoylation [57]. Here we raise the 2nd AR-independent function, which can provide an extra benefit to suppress PCa invasion that neither shAR nor Enz treatment have.

Previous studies have confirmed ATF3 can be activated by ROS in several kinds of human cancer cells [31–33]. Previously our studies demonstrated ASC-J9® treatment can increase endogenous ROS to increase radiation-induced DNA damage [58]. Our present study confirmed ASC-J9® increased ROS can work through inhibiting the expression of GCLC to negatively regulate ROS level.

On the another hand, many PCa studies, when investigating PCa cells invasion, have focused on the MMPs family. In this study, when we investigate the ASC-J9®/ROS/ATF3 pathway, the clinical databases suggest PTK2 has much stronger correlations with PCa metastasis than other known ATF3 related metastasis genes, such as MYC, MMP2, MMP9, and MMP13. This information provides ATF3 as a new candidate for development of new therapeutic strategies.

## Conclusion

Our results suggest that ASC-J9® not only suppresses the PCa progression via degrading AR, but it can also alter the ATF3/PTK2 signaling to suppress the PCa progression in an AR-independent manner. Further understanding and application of ASC-J9® in the treatment of PCa, including AR-dependent and AR-independent anti-cancer effects, can help in the development of novel therapies to better suppress PCa progression.

## Supplementary Information

**Additional file 1:** **Figure S1.** ATF3 knockdown efficiency in C4-2, CWR22Rv1, and PC-3 cell lines. ATF3 expression in C4–2 (left), CWR22RV1 (middle) and PC-3 (right) after knocking down ATF3. Based on ATF3 knocking down efficiency, majority of assay using ATF3-shRNA-2. SCR = Scramble, #1 = ATF3-shRNA-1, #2 = ATF3-shRNA-2.

**Additional file 2:** **Figure S2.** ATF3’s functions in PC-3 cell line. (A) MTT assay using shATF3 indicates suppressing ATF3 could increase cell growth in PC-3 cells. (B) Invasion assay shows using shATF3 to knock down ATF3 increases cell invasion in PC-3 cells. (C) MTT assay indicates oeATF3 in PC-3 cells reduces cell growth. (D) Overexpression of ATF3 (oeATF3) in PC-3 cells can decrease cell invasion (E) MTT analysis shows knock down of ATF3 in PC-3 cells reversed ASC-J9® treatment effects. (F) GSH assay to reveal ASC-J9® can decrease GSH concentration in PC-3 cell. (G) The qRT-PCR of GSH upstream genes GCLC was decreased significantly when treated with ASC-J9® in PC-3 cell. For B and D, quantitations are below or right of images. Data represent the mean ± SD except qRT-PCR represent the mean ± SEM. *p < 0.05, **p < 0.01, by Student t test.

**Additional file 3:** **Figure S3.** (A) Overexpressed ATF3 (oeATF3) could suppress PTK2 expression in PC-3 cells. (B) Western Blots show ASC-J9® treatment could suppress PTK2 expression level in PC-3 cells.

**Additional file 4:** **Figure S4.** IHC image (left) and quantification statistics (right) of Ki67 expression in mice xenograft, magnification, X100, X200. Data represent the mean ± SD, **p < 0.01, *p < 0.05, by one-way ANOVA.

## Data Availability

The analyzed datasets in the study are available from the Chawnshang Chang’s lab on reasonable request.

## Abbreviations

PCa: Prostate cancer
CRPC: Castration-resistant prostate cancer
Enz: Enzalutamide
AR: Androgen Receptor
ATF3: Activating Transcription Factor 3
PTK2: Protein Tyrosine Kinase 2
GCLC: Glutamate-Cysteine lLgase Catalytic
