# Supplementary figures and images for: ASC-J9® suppresses prostate cancer cell proliferation and invasion via altering the ATF3-PTK2 signaling
Source: J Exp Clin Cancer Res. 2021 Jan 4;40:3. doi: 10.1186/s13046-020-01760-2 (PMC7780640; doi:10.1186/s13046-020-01760-2)

Supplementary Figure 1.

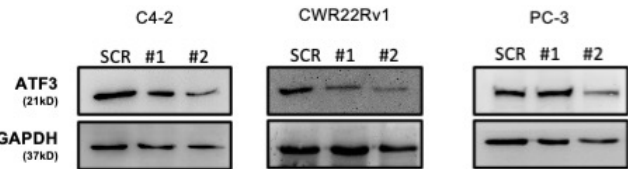

Supplement: Supplementary file 1 — Additional file 1: Figure S1. ATF3 knockdown efficiency in C4-2, CWR22Rv1, and PC-3 cell lines. ATF3 expression in C4–2 (left), CWR22RV1 (middle) and PC-3 (right) after knocking down ATF3. Based on ATF3 knocking down efficiency, majority of assay using ATF3-shRNA-2. SCR = Scramble, #1 = ATF3-shRNA-1, #2 = ATF3-shRNA-2. [file 13046_2020_1760_MOESM1_ESM.pdf]

Supplementary Figure 2.

A.

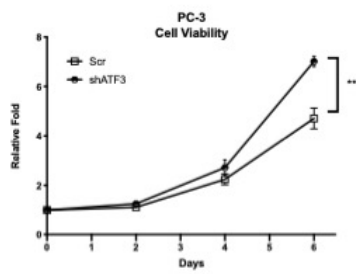

B.

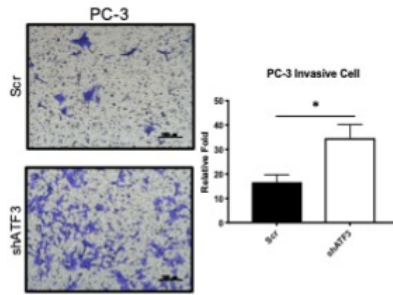

C.

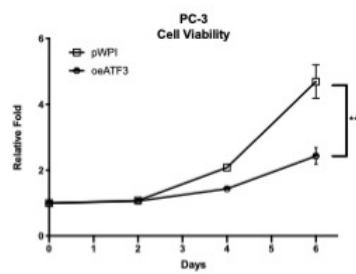

D.

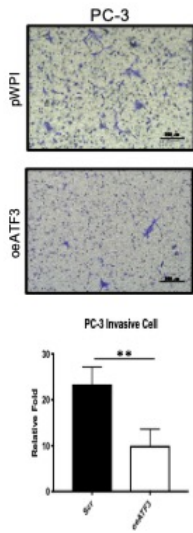

E.

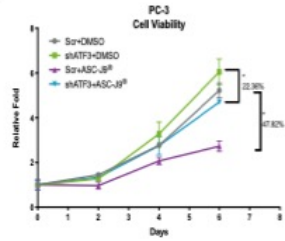

F.

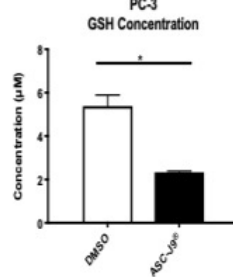

G.

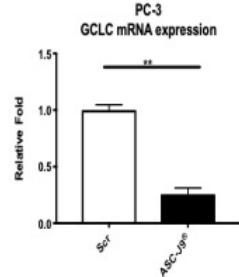

Supplement: Supplementary file 2 — Additional file 2: Figure S2. ATF3’s functions in PC-3 cell line. (A) MTT assay using shATF3 indicates suppressing ATF3 could increase cell growth in PC-3 cells. (B) Invasion assay shows using shATF3 to knock down ATF3 increases cell invasion in PC-3 cells. (C) MTT assay indicates oeATF3 in PC-3 cells reduces cell growth. (D) Overexpression of ATF3 (oeATF3) in PC-3 cells can decrease cell invasion (E) MTT analysis shows knock down of ATF3 in PC-3 cells reversed ASC-J9® treatment effects. (F) GSH assay to reveal ASC-J9® can decrease GSH concentration in PC-3 cell. (G) The qRT-PCR of GSH upstream genes GCLC was decreased significantly when treated with ASC-J9® in PC-3 cell. For B and D, quantitations are below or right of images. Data represent the mean ± SD except qRT-PCR represent the mean ± SEM. *p < 0.05, **p < 0.01, by Student t test. [file 13046_2020_1760_MOESM2_ESM.pdf]

Supplementary Figure 3.

A.

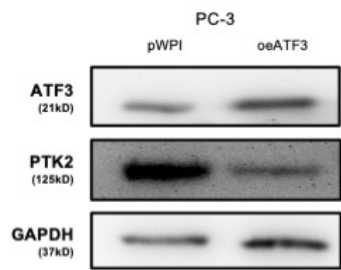

B.

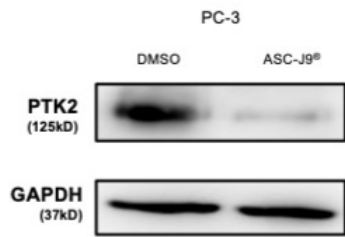

Supplement: Supplementary file 3 — Additional file 3: Figure S3. (A) Overexpressed ATF3 (oeATF3) could suppress PTK2 expression in PC-3 cells. (B) Western Blots show ASC-J9® treatment could suppress PTK2 expression level in PC-3 cells. [file 13046_2020_1760_MOESM3_ESM.pdf]

Supplementary Figure 4.

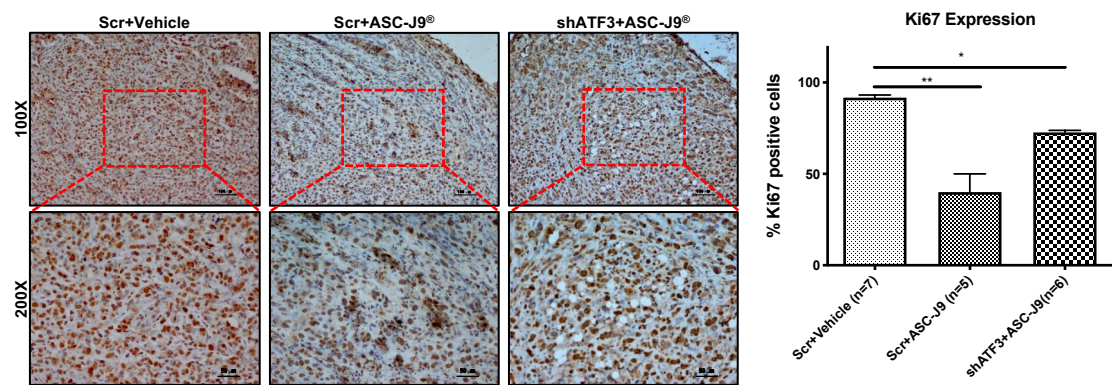

Supplement: Supplementary file 4 — Additional file 4: Figure S4. IHC image (left) and quantification statistics (right) of Ki67 expression in mice xenograft, magnification, X100, X200. Data represent the mean ± SD, **p < 0.01, *p < 0.05, by one-way ANOVA. [file 13046_2020_1760_MOESM4_ESM.pdf]
